# PILOT STUDY OF A SELF-ADMINISTERED ADVANCE PLANNING TOOL FOR TECHNOLOGY USE WITH DEMENTIA CARE DYADS

**DOI:** 10.1093/geroni/igac059.1002

**Published:** 2022-12-20

**Authors:** Clara Berridge, Natalie Turner, Liu Liu

**Affiliations:** University of Washington, Seattle, Washington, United States; University of Washington, Seattle, Washington, United States; University of Washington, Seattle, Washington, United States

## Abstract

We present feasibility and preliminary efficacy findings from a novel web-app intervention to educate and facilitate dyadic communication about four categories of technologies used in dementia care and to document preferences of people living with mild AD regarding these technologies. A pre/post-test design was conducted. Eighty-eight percent of the 66 enrolled participants completed the study for a total of 29 dyads. Participants gave favorable ratings of satisfaction, helpfulness, clarity, and other usability measures. While improvements on multiple measures were of greater magnitude for care partners than for people living with dementia (PLWD), paired t-tests showed statistically significant improvement with medium and large effect sizes in PLWD (p<.001) and care partners’ ( p<.001) self-reported understanding of the technologies, as well as care partners’ perceptions of the PLWD’s understanding (p<.01). After completing the web-app together, care partners felt significantly more prepared to make decisions about these technologies (p<.01).

